# Phosphorus—A Peculiar Symptom

**Published:** 1898-06

**Authors:** F. H. Lutze

**Affiliations:** Brooklyn, N. Y.


### PHOSPHORUS—A PECULIAR SYMPTOM.

F. H. Lutze, M. D., Brooklyn, N. Y.

Mr. F. D. T., æt. 65 years, a real estate agent, called on November 6th, 1893, to be treated for the following symptoms:

Objective: Face sallow, cheeks tinged slightly red.

The "whites of eyes" of a dead white color, lustreless, pupils contracted, vision fairly good.

Ears throughout like yellow wax, translucent.

Subjective: He cannot walk one-half block without being compelled to stand still, hold on to something, and gasp for breath; ascending stairs well-nigh impossible for the same reason; he has to stand still and gasp for breath at every third or fourth step.

Besides this dyspnœa, he has a trembling spell every even-

---

ing after retiring to bed and before sleeping which extends over the whole body. Making a tremulous motion with my hand, I asked him if it was like that, and he answered me yes.

He did not sleep very good after midnight until toward morning.

The appetite is fair, thirst, but drinks only a little at a time; stools and urine normal.

Most of these symptoms had been present for eight or ten years, the trembling spells for at least fifteen years, but all have become worse of late.

He received Ars. 30 or 200 at intervals for three weeks, without any perceptible change, though he imagined that he felt a little better. All the symptoms remained unchanged.

About 8 A. M. December 6th, I received a call to come as quickly as possible to Mr. T., for the family thought him dying.

On my arrival I heard this: Mr. T. had retired to the bathroom, and when he remained there such an unusual long time Mrs. T. went in there, and found him sitting, with body bent forward and vertex of head resting on edge of bathtub, breathless and pulseless as far as she could discover and the face ghastly.

They carried him to a room and placed him on a couch, where he was lying when I arrived, apparently as well as ever.

I concluded that Ars. was not the simillimum, and plied him with questions for a long time to discover symptoms for a new prescription, but in vain. Then turning toward a table to put up some medicine Mrs. T. called: "Doctor, look around quick; he has one of his trembling spells!"

It was not a trembling spell, as I had been led to believe. He was lying on his back, was jerked up violently and fell down again on the back, the motion resembling very much those during sexual intercourse.

This symptom I had seen in Dr. Allen's *Handbook of Meteria Medica* under Phosphorus, Sleep: ". . . and falling asleep, sudden starting up with irrational talking, or during sleep with erotic ecstasies, or lascivious dreams; also with automatic movements as in sexual intercourse."

R. Phos. 200, 4 powders. When he called the next time the improvement was visible, and he received December 15th Phos. 45m., 2 powders.

December 25th. The trembling (jerking) spells have almost entirely ceased; he feels wonderfully improved and looks it. R. Phos. cm., 2 powders.

He did not call again until about six months later. Improved to such an extent that I did not recognize him at first, and he declared himself completely cured and imbued with new life and vigor.

---

